# The Effect of Mandatory Play Breaks on Subsequent Gambling Behavior Among Norwegian Online Sports Betting, Slots and Bingo Players: A Large-scale Real World Study

**DOI:** 10.1007/s10899-021-10078-3

**Published:** 2021-10-12

**Authors:** Niklas Hopfgartner, Michael Auer, Tiago Santos, Denis Helic, Mark D. Griffiths

**Affiliations:** 1 Institute of Interactive Systems and Data Science, Graz University of Technology, Inffeldgasse 16C, 8010 Graz, Austria; 2 Neccton GmbH, Davidgasse 5, 7052 Müllendorf, Austria; 3 International Gaming Research Unit, Psychology Department, Nottingham Trent University, Burton Street, Nottingham, NG1 4BU UK

**Keywords:** Gambling, Responsible gambling, Responsible gambling tools, Problem gambling, Mandatory play breaks, Forced session termination

## Abstract

In order to protect gamblers, gambling operators have introduced a wide range of responsible gambling (RG) tools. Mandatory play breaks (i.e., forced termination of a gambling session) and personalized feedback about the gambling expenditure are two RG tools that are frequently used. While the motivation behind mandatory play breaks is simple (i.e., gambling operators expect gamblers to reduce their gambling significantly as a result of an enforced break in play), empirical evidence supporting the efficacy of the mandatory breaks is still limited. The present study comprised a real-world experiment with the clientele of Norwegian gambling operator *Norsk Tipping*. On the *Norsk Tipping* gambling website, which offers slots, bingo and sports-betting, forced termination occurs if gamblers have played continuously for a one-hour period. The study tested the effect of different lengths of mandatory play breaks (90 s, 5 min, 15 min) on subsequent gambling behavior, as well as the effect of combined personalized feedback concerning money wagered, won, and net win/loss. In total 21,129 online players (61% male; mean age = 47.4 years) experienced at least one play break between April 17 and May 21 (2020) with 156,989 mandatory play breaks in total. Results indicated that a 15-min mandatory play break led to a disproportionately longer voluntary play pause compared to 5-min and 90-s mandatory play breaks. Personalized feedback appeared to have no additional effect on subsequent gambling and none of the mandatory play breaks appeared to affect the increase or decrease in money wagered once players started to gamble again.

## Introduction

In the field of gambling studies, the prevention of problem gambling has become an issue of major concern for gambling operators all around the world. Consequently, in order to protect gamblers and to help them gamble more responsibly, gambling operators have introduced a wide range of responsible gambling (RG) tools. There are many different types of RG tools including various types of limit-setting (deposit limits, spending limits, time limits, etc.), temporary and permanent voluntary self-exclusions, mandatory play breaks, personalized feedback, pop-up messaging, etc. (Harris & Griffiths, 2017).

In a review of online responsible gambling measures, Griffiths (2012) recommended that in those forms of internet gambling that can be played repeatedly (e.g., online slot machine games, online roulette, etc.), online gambling operators should implement a mandatory play break to any player that has been continuously gambling for one hour. He further recommended that the mandatory play break should be for a minimum of five minutes (but preferably longer) and provided two reasons for why this should be the case. The first reason was because gamblers (and particularly problem gamblers) can find it hard to adhere to time and/or money limits they have set themselves while gambling. The second reason is that such breaks, which force gamblers to stop when they have been playing continuously, facilitate ‘time outs’ (i.e., a ‘cooling off’ period) in which gamblers can think more rationally about their gambling behavior. Given that gambling continuously for such long periods of time can also induce dissociative and trance-like states that provide players with psychologically rewarding experiences that facilitate escape from everyday stresses and strains (Griffiths et al., 2006; Wood & Griffiths, 2007), mandatory play breaks may also bring about the end of and/or inhibit such dissociative states.

Furthermore, tolerance and withdrawal play a central role in maintaining an addictive behavior (Mendelson et al., 1998). Griffiths (1993) conducted a study to objectively measure gambling tolerance by examining the excitement levels (measured by heart rate) in regular and non-regular gamblers, and found that regular gamblers experienced a greater reduction in their excitement levels after gambling as compared with non-regular gamblers. Griffiths (1993) hypothesized that this result might provide an objective measure of gambling tolerance, since the decrease in heart rate signified a decrease in the ‘high’ or excitement immediately following gambling, which in turn might cause regular gamblers to gamble faster and more frequently in the future. In addition, the need to gamble with increasing amounts of money to achieve the desired excitement reflects one diagnostic criterion for problematic gambling behavior in the *Diagnostic and Statistical Manual of Mental Disorders* (5th ed.; DSM-5; American Psychiatric Association, 2013). These findings suggest that regular gamblers and in particular problem gamblers become tolerant to gambling due to the reduced effect of the activity.

Having insight into the effectiveness of mandatory play breaks can therefore be helpful for both online gambling and gaming providers, as mandatory play breaks could be an important countermeasure against the development of tolerance. Therefore, it is important to know if the duration of the mandatory play break correlates with craving to play and whether personalized feedback during the mandatory play break has an additional impact on players' behavior. However, there is very little empirical evidence concerning the efficacy of mandatory play breaks especially in relation to how long the mandatory breaks should be. To the best of the present authors’ knowledge, only two studies have ever directly investigated the effects of mandatory play breaks in gambling.

Blaszczynski et al. (2015) carried out a laboratory-based investigation and evaluated the effects of three different lengths of play breaks and their impact on subsequent gambling cravings (assessed using the Gambling Craving Scale Young & Wohl, 2009). The experimental study recruited 141 university students (63 males) who all played a game of simulated electronic blackjack for quarter of an hour (i.e., 15 min). There were three experimental conditions (no play break, 3-min play break, and 8-min play break) and the participants were randomly assigned to one of the three conditions. The study found that self-reported craving among the participants was significantly higher in the 8-min play break condition when compared to participants in the 3-min break condition and those who had no play break. The study also found higher self-reported craving among those in the 3-min play break condition when compared to participants in the no play break condition. The authors also examined participants’ levels of dissociation (utilizing the Dissociative Experience Scale Jacobs, 1988) and found no significant difference between the three experimental groups. However, self-reported craving and self-reported dissociation were positively correlated. Consequently, the results provide some support for the role of dissociation in relation to repeated gambling within a session. The results of the study suggest that there may be unintended consequences of mandatory play breaks and that based on these particular findings alone, mandatory play breaks without any other RG tools might be counterproductive by enhancing urges and cravings to continue. More specifically, mandatory play breaks might have increased efficacy if there is personalized feedback during the mandatory break.

However, as Auer et al. (2019) noted, the study by Blaszczynski et al. (2015) arguably has some major limitations including the ecological validity (i.e., the game was played in a laboratory situation and the blackjack game was simulated with no real money involved rather than being genuine gambling), the small number of participants in each of the three experimental conditions, and the fact that the participants were all university students rather than a group of confirmed gamblers. Auer et al. (2019) also claimed that simulated gambling for just 15 min in a laboratory setting was unlikely to have induced a dissociative state and that an 8-min break (while longer than a 3-min break and no break) may not be a “long” break.

The second empirical study by Auer et al. (2019) investigated the direct effects of mandatory play breaks in gambling among 7190 video lottery terminal (VLT) players from the Norwegian gambling operator *Norsk Tipping*. In this study, VLT gambling sessions which reached a one-hour play duration, led to a forced session termination and a mandatory play break of 90 s. Because the mandatory play break applied to all sessions, the authors had to choose a matched pairs design to analyze the effect of the mandatory play break on subsequent gambling. The authors matched sessions (e.g., those which lasted a few seconds short of one-hour) according to the amount bet and won with terminated sessions and investigated the amount of time that elapsed until players started to gamble again as well as the amount bet after they started to play again in relation to before. The study found that mandatory play breaks led to a shorter voluntary play pause followed by sessions with higher stakes and longer playing duration. Furthermore, gambling expenditure was higher in the subsequent 24 h for sessions with mandatory play breaks. The authors argued that this was likely due to higher intensity gamblers being more likely to trigger mandatory breaks. However, the study’s conclusions were limited due to the lack of an experimental approach because all players who gambled for one hour were forced to take a 90-s break.

There is clearly a lack of empirical studies examining the efficacy of mandatory play breaks and their impact on subsequent gambling behavior. Like the study by Auer et al. (2019), the present study is a real-world investigation using account-based tracking data from a European gambling operator. Account-based behavioral tracking data has been shown to have many advantages compared to survey and experimental data including much larger sample sizes, the sample comprising actual gamblers, and the data being objective (and not susceptible to social desirability or memory recall biases found in self-report data) (Griffiths, 2014).

The present study is the first to examine mandatory play breaks in an online casino setting and the first to follow an experimental approach where players were randomly assigned to the experimental conditions. The authors wanted to test the effect of different lengths of mandatory play breaks (i.e., 90 s, 5 min, 15 min) as well as the effect of combined personalized feedback (i.e., information about money wagered, won, and net win/loss that day). Previous gambling research has demonstrated that personalized messaging can reduce the amount of money that players spend gambling (e.g., Auer & Griffiths, 2015a, 2016, 2020) but that automated pop-up messaging is only effective in stopping individuals gambling in a very small proportion of cases (Auer & Griffiths, 2015b; Auer et al., 2014).

Consequently, to address the lack of a large-scale real-world experiment contributing to the mixed findings of previous research, the present study was designed to answer the following research questions:

(i) Does the length of mandatory play breaks influence subsequent gambling?
(ii) Does personalized feedback during mandatory play breaks influence subsequent gambling?
(iii) How strong are the effects of different lengths of mandatory play breaks in combination with and without personalized feedback on subsequent gambling?

The answers to these questions might have important impacts on technical possibilities in the prevention of gambling disorder.

## Method

### Study Context

The present study was carried out with online players from *Norsk Tipping* (the Norwegian government’s gambling operator). Prior to the study, any player who gambled continuously on the operator’s website for approximately 60 min experienced a mandatory play break of 90 s. The enforced play break is communicated to players via a pop-up message, which simply informs players that they have played continuously for 60 min. The pop-up also tells players that they are unable to gamble for the next 90 s along with a clock counter of the remaining break time before they can gamble again. At the time the study was carried out, *Norsk Tipping’s* online clientele could play a choice of games including bingo, scratch-cards, slots games, and sports betting. In order to gamble on any *Norsk Tipping* game, players have to have personalized accounts. This means that everything they do on the site is automatically tracked and that *Norsk Tipping* has all the information regarding the entire gambling behavior of the individual on that account. It should also be noted that *Norsk Tipping* has a monthly global loss limit so that players cannot lose more than NOK 20,000 a month (approximately US $2000) across all games and that there is a mandatory monthly global loss limit of NOK 10,000 (approximately US $1000) on gambling in their digital channel (online casino, online bingo, and online scratch-cards). In digital channels there is also a daily mandatory loss limit of NOK 4000 (approximately US $400).

### Participants

In total, 21,129 online players experienced at least one play break between April 17 and May 21 (2020). The players average age was 47.4 years (*SD* = 13.53), and 12,894 players were male (61%) and 8235 players were female (39%). The 21,129 players produced 156,989 play break events. Participants were randomly assigned to one of eight groups. There were seven experimental groups and one control group. As longer play breaks could potentially have unforeseeable effects, 60% of participants were assigned to the control group. Participants in the control group did not experience any changes regarding mandatory play breaks. The control group’s play break lasted 90 s and was triggered after one hour of continuous gambling. The remaining participants were randomly assigned to one of the seven experimental groups (details in the next section).

Once players were randomly assigned to one condition, they remained in that condition for the rest of the experimental period. Therefore, players could experience more than one mandatory play break if they played for at least an hour on more than one occasion. Players could not circumvent the play break, even if they opened multiple sessions on their devices.

### Study Design

Between April 17 and May 21 (2020) all players who received at least one play break were assigned to one of eight conditions:

1. CONTROL GROUP: The control group continued to receive the same play break that was already in place prior to the study. After a session of 60 min a pop-up message appeared and informed players that they had just played for 60 min and therefore could not play for the next 90 s. A counter clock displayed the mandatory play break time in seconds remaining until they could gamble again. In the meantime, players could click a button which directed them to *Norsk Tipping’s* responsible gaming site while waiting to gamble again.

For all experimental conditions, an additional logout button was added to the pop-up message concerning the mandatory play break, allowing gamblers to directly log out of their account. To address the first research question, the authors defined the following three groups with different lengths of mandatory play breaks.

2. BREAK 90: This group was identical to the ‘CONTROL GROUP’ condition except that the pop-up message also contained a log-out button.
3. BREAK 300: This group was identical to the ‘BREAK 90’ condition except that after a session of 60 min they could not play again for the next 5 min (300 s).
4. BREAK 900: This group was identical to the ‘BREAK 90’ condition except that after a session of 60 min they could not play again for the next 15 min (900 s).

Regarding the second research question, the authors hypothesized that personalized feedback (i.e., information about the amount bet, won, and the resulting net loss or net win that day) would have an additional beneficial effect on subsequent gambling. Therefore, the following three groups additionally contained personalized feedback as part of the pop-up message of the mandatory play break.

5. FEEDBACK 90: This group was identical to the ‘BREAK 90’ condition except that the pop-up message also informed players about the amount bet, won, and the resulting net loss or net win on that day.
6. FEEDBACK 300: This group was identical to the ‘FEEDBACK 90’ condition except that after a session of 60 min they could not play again for the next 5 min (300 s).
7. FEEDBACK 900: This group was identical to the ‘FEEDBACK 90’ condition except that after a session of 60 min they could not play again for the next 15 min (900 s).

The study also tested the effect of a mandatory play break which did not inform players about the length of the mandatory break that they received after 60 min of continuous play. However, due to customer satisfaction concerns and uncertainty regarding player’s reactions it was decided to only test a 90-s play break duration.

8. NO COUNTDOWN + FEEDBACK 90: After a session of 60 min, a pop-up message appeared and informed players that they had just played for 60 min. The mandatory break lasted for 90 s, but there was no counter clock and players did not know how long the play break would last. The pop-up informed players about the amount bet, won, and the resulting net loss, or net win that day. Players could click a button which directed them to *Norsk Tipping’s* responsible gaming site or a log-out button.

**Fig. 1:**
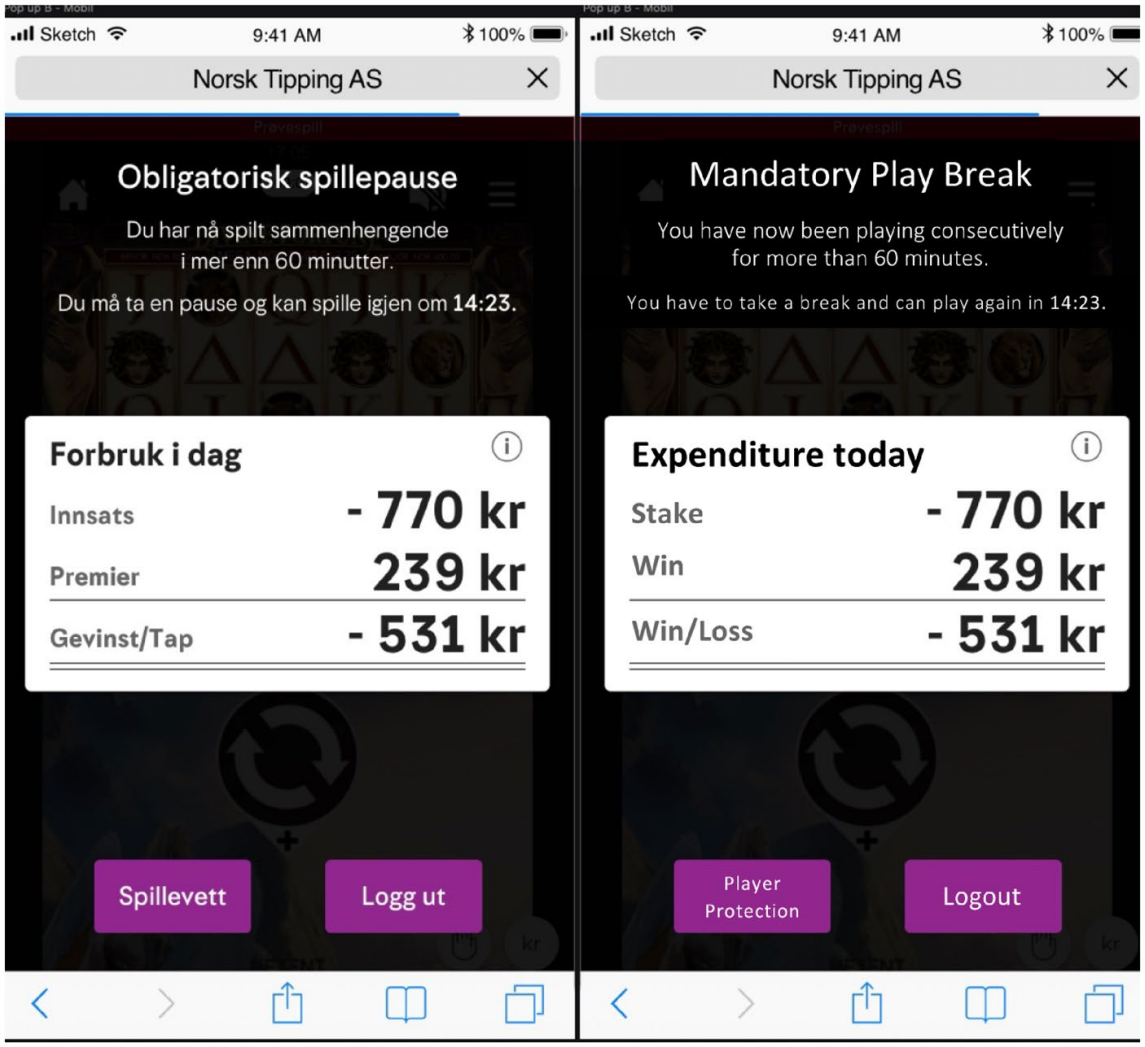
Pop-up window which informed players that they had just played for 60 min and therefore could not play for 90 s/5 min/15 min. The left pop-up shows the original in Norwegian, whereas the right pop-up shows the translation in English. The pop-up also displayed (for four experimental conditions) the amount bet (i.e., stake), won, and net loss/win for that day. The pop-up also contained a button which directed players to *Norsk Tipping’s* Responsible Gaming site and a log-out button

Figure 1 displays the pop-up window as it appeared in the three experimental conditions with personalized feedback (i.e., FEEDBACK 90, FEEDBACK 300, FEEDBACK 900). The dataset comprised each and every game spin, bet, as well as win and the respective timestamp (day, hour, minute, second) of that transaction. The authors developed the following two metrics to analyze the effects of the various mandatory play breaks on subsequent gambling. First, the time between the end of the mandatory play break and the first transaction after the enforced play break was computed. This metric is henceforth referred to as *time to next session* (TTNS). This metric can range from 0 s to days, weeks, or months depending on how frequently or infrequently the player gambles (although the authors only had data available from April 17 to May 21, 2020). Secondly, the amount bet within 60 min once players started to gamble again after the mandatory play break was calculated. Using this amount, the authors calculated the relative change compared to the amount bet within 60 min before the mandatory play break. This metric is henceforth referred to as *relative change in bet* (RCIB).

### Data Analysis

The authors tested whether the TTNS and the RCIB followed a normal distribution according to D’Agostino’s (1971) *K*^2^ test. Both metrics significantly deviated from a normal distribution (*K*^2^ = 467,452, *p* < 0.001 and *K*^2^ = 673,430, *p* < 0.001), therefore non-parametric test statistics were utilized. To investigate whether the length of the mandatory play break and the mandatory play break in combination with personalized feedback had a significant effect on the metrics, the Kruskal Wallis test (Kruskal, 1952) and the Mann–Whitney U test were used (Mann & Whitney, 1947). Finally, a multiple linear regression analysis was performed to measure the effects of mandatory play breaks and personalized feedback. The TTNS formed the dependent variable (DV) and the properties of the conditions (e.g., the length of the play break) the independent variables (IVs). The full linear regression model is shown below:

1 $$\begin{aligned} & \log (TTNS)\sim {\beta}_{0} + {\beta}_{1}\,logout \, button + {\beta}_{2} \,no \, countdown \\ & \quad + \varvec{\beta}_{3}\, mandatory \, break \, duration + {\beta}_{4} feedback \\ & \quad + \varvec{\beta}_{5}\,mandatory \, break \, duration:feedback \\ \end{aligned}$$

The IVs included three binary variables (i.e., *logout button, no countdown, feedback*). The variable *logout button* encoded whether the pop-up message contained a logout button (all conditions except the control group contained a logout button). Therefore, this variable differentiated between the control group and all other conditions. The variable *no countdown* encoded whether the pop-up message did not inform the gambler about the length of the mandatory play break (i.e., condition ‘NO COUNTDOWN + FEEDBACK 90’), whereas *feedback* encoded whether the pop-up message included personalized feedback. Finally, the authors modeled the three different lengths of the mandatory play breaks with a categorical variable (i.e., *mandatory break duration*). Therefore, the bold coefficients represent vectors that hold a scalar coefficient for each categorical value. The authors also included the interaction of *mandatory break duration* and *feedback* in the multiple regression model to measure the additional effect of the personalized feedback during mandatory play breaks. Finally, the authors removed all play break events with a TTNS of zero (i.e., 32 play break events), as well as a TTNS greater than 24 h (i.e., 1,398 play break events) and applied a log-transformation. Due to the design of the regression model the control group is the baseline, which is represented by the intercept of the model given *logout button* = 0, *no countdown* = 0, *feedback* = 0 and *break duration* = ’90 s’. Therefore, the coefficient *ß*_*1*_ represents the relative change in TTNS due to the presence of a logout on the pop-up message of the mandatory play break respectively. Whereas *ß*_*2*_ depicts the effect of the omission of the counter clock in combination with personalized feedback. The coefficient ***ß***_***3***_ holds two values representing the change in TTNS due to a 5-min and 15-min mandatory play break compared to a 90-s break. Finally, the coefficient ***ß***_***5***_ also consists of two values describing the additional effect of personalized feedback in combination with the corresponding mandatory play break. As the TTNS was log-transformed, the exponential function needs to be applied in order to interpret the coefficients with respect to the original distribution of the TTNS. The following transformation was therefore applied: *e*^*β*^−1. It should also be noted that Bonferroni correction was used to compensate for the multiple tests performed in all experiments.

### Ethics

This study was performed in line with the principles of the Declaration of Helsinki. Approval for the study was granted by Nottingham Trent University’s ethics committee.

## Results

**Fig. 2:**
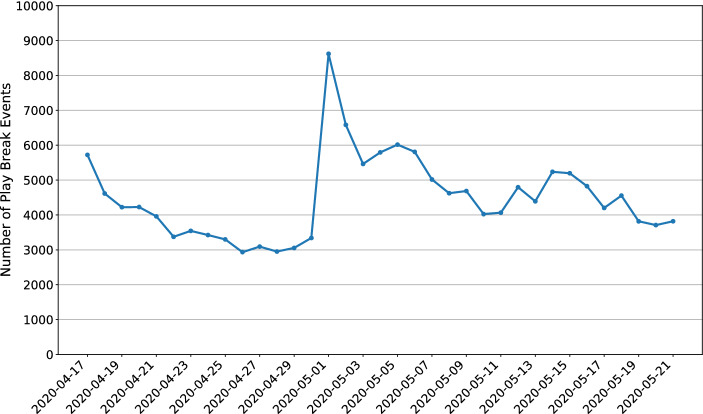
Number of play break events per day over the experimental study period

Figure 2 reports the number of play break events per day. In total, there were 156,989 play break events. The number of events was not evenly distributed, and the highest number was observed on May 1. On average, players experienced 7.43 play breaks (*SD* = 13.8) during the period of study. A quarter of the 21,129 players only received one play break (25%), 50% at most received three play breaks, and 25% experienced at least eight play breaks. There was one player who received 306 play breaks during the study period.

Table 1 reports the number of mandatory play breaks in each experimental group (total of 156,989 play breaks), the median TTNS and RCIB, as well as the 25th and the 75th percentile for both metrics. The median TTNS across all of the 156,989 play breaks was 0.8 min. The shortest median TTNSs were observed in the CONTROL GROUP (0.5 min), BREAK 90 (0.6 min), and the FEEDBACK 90 group (0.6 min). This means that in 50% of the events in these three groups the players started to play within 0.5 to 0.6 min after the 90-s play break was over. The longest TTNSs were observed in the two groups with a 15-min play break (BREAK 900 and FEEDBACK 900). In these two groups, in 50% of the mandatory play breaks, players started to play again within 6.5 and 6.7 min after the break was over, respectively. In the group where players were not informed about the length of the mandatory play break (NO COUNTDOWN + FEEDBACK 90), the median TTNS was 1.0 min. Using the Kruskal–Wallis test, analysis showed a significant difference in the TTNS between the eight groups (*H* = 17,653.36, *p* < 0.001). The median RCIB across all groups was -20%, which means that there was a median reduction in amount bet of 20%. There was a significant difference in RCIB between the eight groups (*H* = 45.66, *p* < 0.001).

**Table 1:** Number of play break events in each group and median, 25th, and 75th percentile of the time to next session (TTNS) in minutes and the relative change in bet (RCIB) in percent for each group

Group	N	%	Median TTNS	P25, P75 TTNS	Median RCIB	P25, P75 RCIB
CONTROL GROUP	88,727	57	0.5	[0.1, 2.2]	− 20.4	[− 60.7, 18.6]
NO COUNTDOWN + FEEDBACK 90	10,075	6	1.0	[0.2, 4.0]	− 22.6	[− 62.5, 17.7]
BREAK 90	10,009	6	0.6	[0.1, 3.0]	− 19.2	[− 60.0, 20.1]
BREAK 300	9889	6	1.6	[0.4, 9.1]	− 19.1	[− 58.4, 19.9]
BREAK 900	9292	6	6.5	[1.2, 39.9]	− 17.3	[− 56.7, 21.2]
FEEDBACK 90	10,323	7	0.6	[0.1, 2.6]	− 20.0	[− 61.9, 17.5]
FEEDBACK 300	9927	6	1.6	[0.4, 8.7]	− 19.6	[− 58.7, 20.4]
FEEDBACK 900	8747	6	6.7	[1.2, 45.5]	− 19.6	[− 60.0, 19.2]
	156,989	100				

Regarding the first research question, the TTNS in the BREAK 90 group was significantly smaller than in the BREAK 300 group (*U* = 36,630,284, *p* < 0.001), and the TTNS in the BREAK 300 group was also significantly smaller than in the BREAK 900 group (*U* = 32,816,276, *p* < 0.001). Similarly, the TTNS in the FEEDBACK 90 group was significantly smaller than in the FEEDBACK 300 group (*U* = 36,842,038, *p* < 0.001), and the TTNS in the FEEDBACK 300 group was also significantly smaller than in the FEEDBACK 900 group (*U* = 30,657,475, *p* < 0.001). In terms of RCIB, there was no significant difference between the two groups BREAK 90 and BREAK 300 (*U* = 49,268,331, *p* = 0.29), as well as between the BREAK 300 and BREAK 900 groups (*U* = 45,107,220, *p* = 0.014^1^). Similarly, there was no significant difference between the two groups FEEDBACK 90 and FEEDBACK 300 (*U* = 50,313,783, *p* = 0.013^1^), as well as the FEEDBACK 300 and FEEDBACK 900 groups (*U* = 43,237,217, *p* = 0.31).

The present study further tested the effect of personalized feedback (i.e., information about the amount of money bet, won, and lost) in combination with mandatory play breaks on the TTNS and RCIB. Regarding the TTNS, there was no significant difference between the two groups BREAK 90 and FEEDBACK 90 (*U* = 51,083,151, *p* = 0.08), BREAK 300 and FEEDBACK 300 (*U* = 48,894,320, *p* = 0.32), and BREAK 900 and FEEDBACK 900 (*U* = 40,250,182, *p* = 0.13). Similarly, there was no significant difference in terms of RCIB for the two groups BREAK 90 and FEEDBACK 90 (*U* = 50,828,745, *p* = 0.023^2^), and BREAK 300 and FEEDBACK 300 (*U* = 48,942,914, *p* = 0.36). Only in group FEEDBACK 900 the RCIB was significantly smaller than in the BREAK 900 group (*U* = 39,619,577, *p* < 0.008333^2^).

Table 2 reports the findings of the multiple linear regression model (Eq. 1). The adjusted *R*^2^ value is 0.115 (*F* = 2,878, *p* < 0.001), which means that approximately 11.5% of the variation in the TTNS was explained by the independent variables of the regression model. The transformed regression coefficients (*e*^*β*^−1) represent the relative change in TTNS compared to the control group. Overall, gamblers significantly increased their TTNS by 24% due to the availability of a logout button in the pop-up message of the mandatory play break (c.f. coefficient for variable *logout button*). The removal of the clock counter and therefore not informing gamblers about the length of a 90-s mandatory play break in combination with personalized feedback and a logout button also led to a significant increase in the TTNS by 66% (c.f. coefficient for variable *no countdown*). Increasing the length of the mandatory play break to five and 15 min led to a significant increase in the TTNS of 182% and 809% respectively (c.f. coefficients for variables *break duration* [5 min] and *break duration* [15 min]). Personalized feedback in combination with the mandatory play break did not significantly change the TTNS, which is in line with the non-significant result of the Kruskal Wallis test. Therefore, the coefficients for the regression variables *feedback* as well as the interaction of *feedback* and *break duration* were not significant. Finally, the same multiple linear regression as in Eq. 1 with RCIB as the dependent variable yielded no significant coefficients with an adjusted *R*^2^ < 0.0001.

**Table 2:** Multiple linear regression analysis for the time to next session (TTNS)

Variable	*β*	Std. error	*t*	*e*^*β*^–1	CI (*e*^*β*^–1)
Intercept	−0.4445***	0.008	−58.563	−0.359	[−0.372, −0.346]
*Logout button* [True]	0.2149***	0.024	9.007	0.240	[0.163, 0.322]
*No countdow*n [True]	0.5070***	0.032	15.997	0.660	[0.524, 0.808]
*Feedback* [True]	−0.0486	0.032	−1.530	−0.047	[−0.125, 0.038]
*Break duration* [5 min]	1.0353***	0.032	32.222	1.816	[1.583, 2.070]
*Break duration* [15 min]	2.2071***	0.033	67.443	8.089	[7.323, 8.925]
*Break duration* [5 min]: *Feedback* [True]	0.0426	0.045	0.941	0.044	[−0.076, 0.179]
*Break duration* [15 min]: *feedback* [True]	0.1067	0.046	2.296	0.113	[−0.018, 0.261]
Adjusted *R*^2^				0.115	
Number of observations				155,559	

The number of stars indicate the size of *p*-values, ***: *p* < 0.001, **: *p* < 0.01, *: *p* < 0.05

## Discussion

The present experimental, real-world study investigated the effect of a forced online gambling session termination after 60 min followed by a 90-s, 5-min, or 15-min mandatory play break on subsequent gambling behavior. Furthermore, the effect of personalized feedback (i.e., information about the amount of money bet, won, and net win/loss that day) in combination with a mandatory play break was also investigated. In one experimental condition players were not told how long a 90-s play break would last.

The number of mandatory play breaks per day was not evenly distributed throughout the experimental period. Figure 2 shows that the number of events declined from April 17 to April 30 and was much higher on May 1 than on any other day. From May 1, it again declined until May 21. This is in part due to the fact that *Norsk Tipping’s* players have mandatory limits as well as their own personal limits. Gambling-intense players usually spend the maximum amount allowed by *Norsk Tipping* at the beginning of the month which explains the spike in the number of events on May 1, when gambling-intense players who were unable to play on April 17 (because they had reached their monthly spending limit) returned. This is also evidence that the whole spectrum (low to high intensity gamblers) was part of the study.

The duration between the end of a 90-s, 5-min, or 15-min play break until the start of the next gambling session was measured. This is an indication of the effectiveness of the mandatory play break. A longer time to next session (TTNS) indicates that the mandatory play break was able to stop the player from gambling and possibly bring to an end a state of dissociation if the player was experiencing one (Griffiths et al., 2006; Monaghan, 2009). The distribution of the TTNS significantly deviated from a normal distribution, which is to be expected. Barabasi (2005) argues that many human activities, ranging from communication to entertainment and work patterns, follow non-Poisson statistics, characterized by bursts of rapidly occurring events separated by long periods of inactivity which leads to heavily skewed distributions.

In the context of the present study, gambling is a task with a high perceived priority, since players gambled continuously for 60 min. Therefore, most gamblers start to play again rapidly after the mandatory play break, whereas a few conduct long pauses before they start to gamble again. The longest median TTNS was observed for the two 15-min mandatory play breaks. Whereas all other six conditions had a median TTNS of less than two minutes, the two 15-min play breaks led to a median TTNS of 6.5 and 6.7 min, respectively. The difference between the 15-min play breaks compared to the shorter play breaks is most evident among players at the 75th TTNS percentile. Here, 25% of players waited for at least 39.9 and 45.5 min (respectively) until they gambled again. Overall, it appears that the length of the TTNS increases with the length of the mandatory play break. However, there appears to be a disproportionate difference between a 90-s/5-min play break, compared to 15-min. The effect of a 15-min play break has not been empirically studied previously.

Several real-world gambling studies have shown the effectiveness of bespoke personalized feedback concerning gambling expenditure on subsequent gambling (e.g., Auer & Griffiths, 2015a, 2018, 2020). Also, laboratory studies have shown significant effects of personalized feedback on subsequent gambling behavior (e.g., Neighbors et al., 2015; Wohl et al., 2013). Consequently, it is surprising that there was no significant effect of personalized feedback in combination with mandatory play breaks in the present study. There only appeared to be a difference with respect to the 15-min condition. In Table 1, the median TTNS was larger in the condition FEEDBACK 900 (6.7 min) compared to the condition BREAK 900 (6.5 min). The difference appeared to be even more prominent with respect to the 75th percentile of the TTNS (45.5 vs. 39.9 min). However, the difference was not statistically significant.

The difference with respect to TTNS between the experimental groups was further analyzed using a multiple regression model. The regression model’s effects were coded for the control group to be the baseline, which means that each variable’s effect can be interpreted in relation to the control group. It suggested that the existence of a log-out button led to a significant longer play break. This is in line with previous research, which found that an interactive component increases the adherence to a personal session limit (Wohl et al., 2014). The regression model also reported that players who did not know how long the play break would last, had a longer TTNS. This is to be expected, because players did not know when they were going to be able to gamble again. However, it can be assumed that players would find out at some point of time if the mandatory play break would always be the same as the experimental period progressed. The multiple regression further complemented the Kruskal Wallis tests and found that the 15-min mandatory play break led to a significantly longer TTNS compared to the control group. The same holds true for the five-minute mandatory play break compared to the control group. The only non-significant effects in the multiple regression model were related to the personalized feedback. Neither the coefficient for the feedback variable nor the coefficients for the interaction between feedback and play break duration were significant.

One reason for the non-significant effect of personalized feedback could be the mode of presentation or the design of the pop-up. Wohl et al., (2013, 2014) found that responsible gaming tools which followed human–computer-interaction principles and persuasive system design were more effective. Future studies should further investigate the effect of personalized feedback in combination with mandatory play breaks by changing the way the message is presented. Future studies could also test the effect of normative feedback in combination with mandatory play break pop-ups.

Blaszczynski et al. (2015) reported increased self-reported craving after longer mandatory play breaks and Auer et al. (2019) reported increased wagering after forced VLT session terminations. For that reasons the present study also investigated if longer mandatory play breaks led to increased gambling afterwards. The authors calculated the relative change of the bet (RCIB) in the 60 min after the play break compared to the amount bet within 60 min before the mandatory play break. Longer play breaks neither led to increased gambling, nor to decreased gambling. This further supports the efficacy of longer play breaks on the interruption of long gambling sessions. There was also no effect of personalized feedback on the intensity of gambling immediately after the play-break, except in the BREAK 900 group, which reduced the amount bet slightly, but significantly less (*Mdn* = −17.3%) than the FEEDBACK 900 group (*Mdn*= −19.6%—i.e., median RCIB of the two groups BREAK 900 and FEEDBACK 900 in Table 1). However, this finding was not supported by the results of the multiple linear regression, as none of the coefficients were significant. One explanation for this small effect could be slight differences in the population characteristics due to the unequal assignment of the control and experimental groups. Therefore, additional research is required to further investigate personalized feedback in combination with mandatory play breaks.

The median RCIB was negative for each of the eight conditions which means that in 50% of the mandatory play breaks, players wagered less money after the play break compared to before. One reason that all conditions experienced a reduction in amount bet after the mandatory play break could be the regression toward the mean (Nesselroade et al., 1980). The regression towards the mean explains the fact that extreme events tend to be followed by less extreme events. In the present study, breaks tended to interrupt extreme gambling sessions. Therefore, the subsequent gambling session was more likely to be less extreme than more extreme.

Although the present study does not contain self-report data on craving to gamble and therefore craving can only be inferred from the observed data, it does not appear that a longer play break leads to increased craving which would most likely lead to a shorter TTNS. Increased craving would most likely lead to higher wagers after a mandatory play break. This contradicts the (laboratory-based) findings of Blaszczynski et al. (2015) who concluded that self-reported craving was higher after an eight-minute play break compared to a three-minute play break or no play break. However, their study was not based on player tracking data and the playing duration before the play break was only 15 min which was only one-quarter of the 60-min duration in the present study.

### Limitations

Although the present study had a large sample size and comprised objective account-based data, it is not without limitations. As with most other studies using behavioral tracking data, online gambling data were collected from a single gambling operator (i.e., *Norsk Tipping*) and all the gamblers were Norwegian citizens. Therefore, the findings cannot necessarily be generalized, and future studies should replicate the study here using different operators from different countries. Another limitation is that most players on the website had been subject to a 90-s mandatory play break before the experimental period and therefore already had some experience with this particular responsible gaming tool. It cannot be ruled out that mandatory play breaks would affect players differently if they were presented in a different way or if the pop-up message conveyed different information or was designed differently. Auer and Griffiths (2015b) showed that references to responsible gambling tools and normative feedback increased the number of online players who voluntarily decreased their gambling and/or stopped to play after seeing a pop-up message. Future studies should test different lengths of session before they are forcibly terminated (e.g., after 30 min, 45 min, or longer than 60 min) and different lengths of play breaks not investigated here (e.g., longer breaks such as 30 min).

## Conclusions

The present study adds to the few empirical studies that have evaluated the impact of mandatory play breaks on subsequent gambling behavior. Based on the findings here, it appears that a 15-min play break leads to a disproportionately longer voluntary play pause compared to a 90-s break or 5-min break. Consequently, 15-min mandatory play breaks appear to interrupt players gambling significantly more than a 90-s or 5-min mandatory play break. The study also showed that longer play breaks did not lead to increased wagering and therefore supports the efficacy of a 15-min mandatory play breaks as a way to reduce the intensity of gambling.

The underlying study could have significant implications on online gambling policies. Regulators are increasingly demanding that operators interact with high intensity gamblers and provide evidence of the impact of the interaction on subsequent gambling behavior. Only a few regulators internationally require mandatory play breaks. However, most operators inform players about long gambling sessions, but they do not interrupt them. Findings in the present study could provide important evidence for regulators to argue the incorporation of 15-min mandatory play breaks on online-casino sites. Although the present study was conducted with online gamblers the results could potentially also lead to further research in the area of internet gaming disorder. To the authors knowledge, internet gaming providers do not routinely inform players about long gaming sessions, nor do they use enforced play breaks. Consequently, this could be an important tool in the prevention of internet gaming disorder.

## Footnotes

1 Eight statistical tests were performed to test the hypothesis of whether mandatory play breaks influenced subsequent gambling. Therefore, statistical significance was considered at the Bonferroni-corrected *p*-value of 0.05/8 = 0.00625.

2 Six statistical tests were performed to test the hypothesis of whether personalized feedback had an additional effect on subsequent gambling. Therefore, statistical significance was considered at the Bonferroni-corrected *p*-value of 0.05/6 = 0.008333.

## References

[CR1] Auer M, Griffiths MD (2015). The use of personalized behavioral feedback for online gamblers: an empirical study. Frontiers in Psychology.

[CR2] Auer M, Griffiths MD (2015). Testing normative and self-appraisal feedback in an online slot-machine pop-up message in a real-world setting. Frontiers in Psychology.

[CR3] Auer M, Griffiths MD (2016). Personalized behavioral feedback for online gamblers: a real world empirical study. Frontiers in Psychology.

[CR4] Auer M, Griffiths MD (2018). Cognitive dissonance, personalized feedback, and online gambling behavior: an exploratory study using objective tracking data and subjective self-report. International Journal of Mental Health and Addiction.

[CR5] Auer M, Griffiths MD (2020). The use of personalized messages on wagering behavior of Swedish online gamblers: an empirical study. Computers in Human Behavior.

[CR6] Auer M, Malischnig D, Griffiths MD (2014). Is ‘pop-up’ messaging in online slot machine gambling effective as a responsible gambling strategy? An empirical re-search note. Journal of Gambling Issues.

[CR7] Auer M, Hopfgartner N, Griffiths MD (2019). The effects of a mandatory play break on subsequent gambling among Norwegian video lottery terminal players. Journal of Behavioral Addictions.

[CR8] Barabasi AL (2005). The origin of bursts and heavy tails in human dynamics. Nature.

[CR9] Blaszczynski A, Cowley E, Anthony C, Hinsley K (2015). Breaks in play: do they achieve intended aims?. Journal of Gambling Studies.

[CR10] D’Agostino RB (1971). An omnibus test of normality for moderate and large sample size. Biometrika.

[CR11] Griffiths M (1993). Tolerance in gambling: an objective measure using the psychophysiological analysis of male fruit machine gamblers. Addictive Behaviors.

[CR12] Griffiths MD, Williams R, Wood R, Parke J (2012). Internet gambling, player protection and social responsibility. Routledge handbook of internet gambling.

[CR13] Griffiths MD (2014). The use of behavioural tracking methodologies in the study of online gambling. SAGE Research Methods Cases.

[CR14] Griffiths MD, Wood RTA, Parke J, Parke A, Allcock C (2006). Dissociative states in problem gambling. Current issues related to dissociation.

[CR15] Harris A, Griffiths MD (2017). A critical review of the harm-minimisation tools available for electronic gambling. Journal of Gambling Studies.

[CR16] Jacobs D (1988). Evidence for a common dissociative-like reaction among addicts. Journal of Gambling Behavior.

[CR17] Kruskal WH (1952). A nonparametric test for the several sample problem. The Annals of Mathematical Statistics.

[CR18] Mann HB, Whitney DR (1947). On a test of whether one of two random variables is stochastically larger than the other. The Annals of Mathematical Statistics.

[CR19] Mendelson JH, Sholar M, Mello NK, Teoh SK, Sholar JW (1998). Cocaine tolerance: behavioral, cardiovascular, and neuroendocrine function in men. Neuropsychopharmacology.

[CR20] Monaghan S (2009). Responsible gambling strategies for internet gambling. The theoretical and empirical base of using pop-up messages to encourage self-awareness. Computers in Human Behavior.

[CR21] Neighbors C, Rodriguez LM, Rinker DV, Gonzales RG, Agana M, Tackett JL, Foster DW (2015). Efficacy of personalized normative feedback as a brief intervention for college student gambling: A randomized controlled trial. Journal of Consulting and Clinical Psychology.

[CR22] Nesselroade JR, Stigler SM, Baltes PB (1980). Regression toward the mean and the study of change. Psychological Bulletin.

[CR23] American Psychiatric Association. (2013). Diagnostic and statistical manual of mental disorders (5th ed.). doi: 10.1176/appi.books.9780890425596

[CR24] Wohl MJ, Gainsbury S, Stewart MJ, Sztainert T (2013). Facilitating responsible gambling: the relative effectiveness of education-based animation and monetary limit setting pop-up messages among electronic gaming machine players. Journal of Gambling Studies.

[CR25] Wohl MJ, Parush A, Kim HAS, Warren K (2014). Building it better: applying human–computer interaction and persuasive system design principles to a monetary limit tool improves responsible gambling. Computers in Human Behavior.

[CR26] Wood RTA, Griffiths MD (2007). A qualitative investigation of problem gambling as an escape-based coping strategy. Psychology and Psychotherapy: Theory, Research and Practice.

[CR27] Young MM, Wohl MJA (2009). The gambling craving scale: psychometric validation and behavioural outcomes. Psychology of Addictive Behaviors.

